# Fostering diagnostics of cardiovascular disease in diabetics using ABI and baPWV – correspondence

**DOI:** 10.1097/MS9.0000000000000370

**Published:** 2023-03-27

**Authors:** Sadia Manan, Omer Ahmed Shaikh, Gulrukh Shaikh, Kanza Saleem, Irfan Ullah, Muhammad Sohaib Asghar

**Affiliations:** a Ziauddin Medical University; b Department of Medicine, Liaquat National Hospital and Medical College, Karachi, Pakistan; c Kabir Medical College, Gandhara University, Peshawar, Pakistan; d Division of Nephrology and Hypertension, Mayo Clinic, Rochester, Minnesota, USA

The prevalence of diabetes mellitus (DM), which affects people of all socioeconomic backgrounds, continues to climb at an alarming rate. Diabetes affects nearly half a billion people worldwide, and it is anticipated that this statistic will soar by more than 50% over the upcoming quarter of a century. Because of how ubiquitous it is all throughout the world, DM has become a cause of massive concern for the population’s health. People who have diabetes have a higher risk of developing chronic conditions that are linked to damage to either the microvascular or macrovascular systems. Complications involving the lower extremities, such as peripheral artery disease (PAD), are becoming more prevalent worldwide. This has a substantial impact on the mortality and morbidity rates of diabetic patients^1^.

PAD is known as the narrowing or occlusion of the arteries in the lower extremities as a consequence of atherosclerosis. Some of the most prominent atherosclerotic risk factors for the development of PAD include smoking, DM, increasing age, and dyslipidemia. DM has been associated with a two-fold to four-fold increase in the incidence of PAD. Undeterred by the fact that PAD remains significant both epidemiologically and clinically, it still falls prey to the vicious cycle of being unattended, underdiagnosed and thus, undertreated. This may be due to the fact that a mere 10% of the patients exhibit typical symptoms of the disease^2^.

The ankle-brachial index (ABI) is a valuable test for assessing atherosclerotic disease of the lower extremities. This test evaluates a decline in the systemic arterial pressure of the lower limb. Patients who have aberrant ABI values, that is either less than 0.9 or greater than 1.3, have an increased likelihood of suffering from cardiovascular events^3^.

In Asia, in particular, the brachial-ankle pulse wave velocity (baPWV) is frequently used owing to its benefits of ease of use, high reproducibility, and significant correlation with aortic PWV. A high baPWV is linked to an elevated risk of all-cause and cardiovascular mortality^4^. To determine the baPWV, the distance that separates the brachial and tibial arteries is divided by the amount of time that a pulse wave takes to travel between these two arteries^5^.

Both baPWV and ABI are prominent noninvasive indicators that are utilized in the process of diagnosing atherosclerosis. In a recent study carried out in Taiwan, Lin *et al*. intended to determine the cumulative, interactive, and independent correlations of ABI and baPWV with mortality from all-cause and expanded cardiovascular disease in type 2 DM patients. The results of this study disclosed that ABI and baPWV are critical determinants that can assist in predicting all-cause and expanded cardiovascular disease mortality in type 2 DM patients and in identifying high-risk patients who would benefit from diabetes therapy in comparison to ABI or baPWV alone. This practical approach has the potential to be implemented into mainstream clinical practices in order to customize risk assessment and direct clinical decisions^5^.

## Ethical statement

Not applicable.

## Conflicts of interest disclosure

The authors declare no conflicts of interest.

## Provenance and peer review

Externally peer-reviewed, not commissioned.
